# Metagenomic Insights of the Root Colonizing Microbiome Associated with Symptomatic and Non-Symptomatic Bananas in *Fusarium* Wilt Infected Fields

**DOI:** 10.3390/plants9020263

**Published:** 2020-02-18

**Authors:** Manoj Kaushal, George Mahuku, Rony Swennen

**Affiliations:** 1 International Institute of Tropical Agriculture (IITA), Mikocheni B, Dar es Salaam-34441, Tanzania; g.mahuku@cgiar.org; 2 Bioversity International, Willem De Croylaan 42, B-3001 Leuven, Belgium; R.Swennen@cgiar.org; 3 Laboratory of Tropical Crop Improvement, Division of Crop Biotechnics, KU Leuven, B-3001 Leuven, Belgium; 4 International Institute of Tropical Agriculture. c/o The Nelson Mandela African Institution of Science and Technology (NM-AIST), P.O. Box 447, Arusha 23306, Tanzania

**Keywords:** banana, microbial diversity, rhizosphere, metagenomics, *Fusarium oxysporum*

## Abstract

Plants tissues are colonized by diverse communities of microorganisms called endophytes. They are key determinants of plant production and health, for example by facilitating nutrient exchanges or limiting disease development. Endophytic communities of banana plants have not been studied until very recently, and their potential role in disease development has not been explored so far. Roots from symptomatic and non-symptomatic banana plants were sampled from fields infected by *Fusarium oxysporum* f.sp. *cubense* race 1. The goal was to compare the endophytic microbiota between symptomatic and non-symptomatic plants through high throughput sequencing of 16s rDNA and shotgun metagenome sequencing. The results revealed that the endophytic root microbiome in bananas is dominated by Proteobacteria and Bacteroidetes followed to a lesser extent by Actinobacteria. The development of disease greatly impacted the endophytic microbial communities. For example, Flavobacteriales abundance was correlated with symptom development.

## 1. Introduction

Plants live in close relationship with microbes that inhabit the rhizosphere region. The microbiome plays a key role for plant health and productivity via nutrient acquisition, tolerance to stress conditions, and inducing resistance against phytopathogens [1]. In addition, plants recruit microflora via the production of root exudates. The endophytic microbiome corresponds to the microbes that colonize and reside within the plant tissues [2]. These microbiomes have been exploited as biocontrol agents promoting plant growth and health in various crops [3,4]. Despite this, endophytic microbiota are poorly studied. Advanced technologies such as high throughput sequencing (HTS) allow for an in-depth characterization of the functions of the plant endophytes [5,6,7].

Bananas contribute to a major portion of the diet and a key source of the income for small holder farmers in Sub-Saharan Africa (SSA). Mchare is an important highland cooking banana, especially in the highlands of Northern Tanzania and Great Lake regions of Kenya and Uganda [8], and Sukari Ndizi is a popular dessert banana in Eastern Africa [9]. Banana plants are multiplied clonally through suckers in the field or in a nursery (macropropagation) or by in vitro culture (micropropagation). The production of bananas has been limited by biotic and abiotic causes [10]. Various insects, pests, and diseases constitute the biotic constraints to the banana plants, whereas the abiotic constraints include heat, desiccation, salt, nutrient imbalance, and physical stress such as wind. *Fusarium* wilt (FW), caused by the soil-borne fungus *Fusarium oxysporum* f. sp. *cubense* (Foc) is one of the most devastating diseases [11]. In SSA, banana production is constrained by *Fusarium* wilt due to Foc race 1. In addition, there is a threat posed by Foc race 4 [12,13,14,15], which is only found in Mozambique but which could emerge in other countries [16] due to movement of planting materials. Foc invades the vascular tissues of bananas through the roots and corm. The initial symptoms correspond to leaves discolouration and wilting, which eventually turn into a bright yellow colour and dead leaf margins. In the advanced stages of infection, more leaves become yellow, and necroses (brown lines) are evident inside the pseudo-stem. The spores of Foc can persist in the soil for decades in the absence of a host. Besides resistant varieties or disease-free planting material, no other effective disease control methods exist for Foc [17]. Mchare and Sukari Ndizi are the most preferred varieties by smallholder farmers in Tanzania that are susceptible to Foc race 1. Nevertheless, susceptible banana plants in East African fields, cultivated with other banana varieties in association with other crops, can remain non-symptomatic while neighbouring banana plants exhibit symptoms and succumb. This is in contrast with large monocultured commercial plantations which are entirely wiped out once Foc has entered. This phenomenon deserves a better characterization as these plants were clonally multiplied and are growing in the same pedo-climatic conditions. Such fields were identified in Tanzania, and we hypothesize that plant microbiota preserve the plant health and limit the disease development. More specifically, part of the endophytic microbiota would colonize the inner tissues of the host and could be in direct contact with Foc.

The level of microbiome diversity was directly correlated with the resistance to the Foc invasions in bananas [18] and multiplication in the roots. Sometimes, host plants also recruit specific beneficial microbiota after phytopathogen infections, which helps the plants to resist and withstand the diseases caused by these pathogens [19]. We aimed to understand the composition and distribution of the microbiome in symptomatic and non-symptomatic banana roots in two locations and in two cultivars. We also aimed to analyse the banana root microbiome of Foc symptomatic samples using sequencing-based functional metagenomics to characterize the gene repertoire of the banana endophytes.

## 2. Results

### 2.1. General Characteristics of the Amplicon

After QIIME filtration and the removal of chimeric sequences, 308,784 seq out of 349,670 (S1H), 337,074 seq out of 381,417 (S2I), 242,504 seq out of 278,606 (S3H), 281,162 seq out of 307,503 (S4I), 464,508 seq out of 544,149 (S5H), 385,006 seq out of 457,699 (S6I), 199,861 seq out of 222,216 (S7H), and 414,864 seq out of 467,607 (S8I) were obtained and taken forward for analysis (Supplementary Table S1). Illumina sequencing for the pooled symptomatic samples resulted in a total of 99,692,954 high quality reads (average length of 312 bp) and about 13.97 Gb of base pairs from a mate-pair library. The high-quality reads were assembled de novo with metaSPAdes, followed by gene prediction using Prodigal at the metagenome mode wherein a total of 573,271 genes were identified.

### 2.2. Root Associated Microbiome Composition and Structure in Symptomatic and Non-Symptomatic Banana

**Figure 1:**
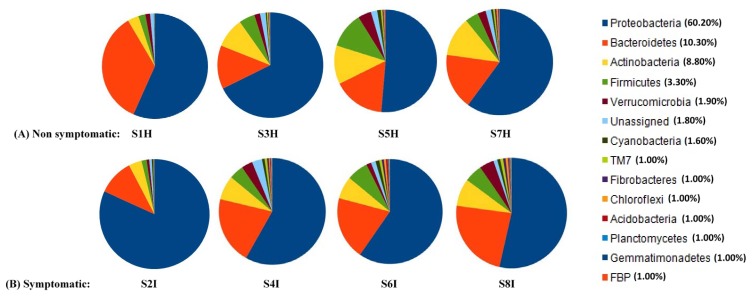

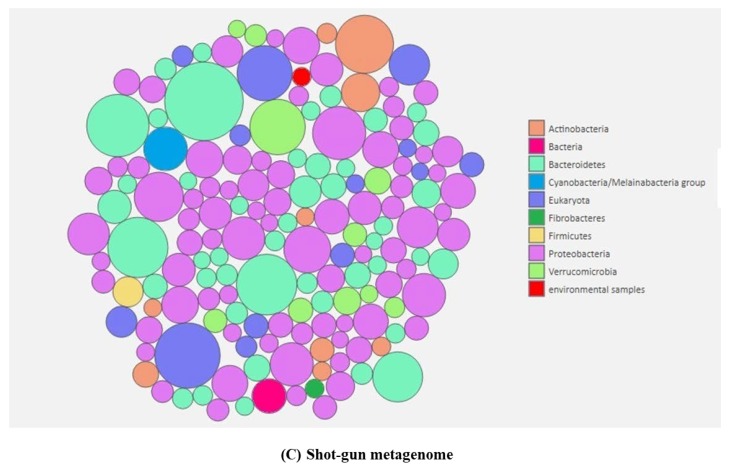
The composition and relative abundance of major bacterial taxa of the root-associated microbiome in non-symptomatic and symptomatic banana. (**A**) Endophytes inhabiting the non-symptomatic banana roots, (**B**) Endophytes inhabiting the symptomatic banana roots, and (**C**) Community composition. Where: *S: Sample; 1–8: Number; H: Non-symptomatic; I: Symptomatic. Samples from TACRI: S1H, S2I (Mchare); S3H, S4I (Sukari Ndizi), and samples from Akheri Kati: S5H, S6I (Mchare); S7H, S8I (Sukari Ndizi).

After removal of singleton operational taxonomic units (OTUs), 21,897 OTUs were clustered with a variable repartition among samples: 6571 (S1H), 5561 (S2I), 6782 (S3H), 5311 (S4I), 7450 (S5H), 8747 (S6I), 4867 (S7H), and 7811 (S8I) (Supplementary Table S2). The closely related microbiota were found to be higher in symptomatic samples rather than in non-symptomatic root samples at both locations. The results revealed that Proteobacteria were the predominant bacterial groups in the non-symptomatic samples, followed by Bacteriodetes and Actinobacteria (Figure 1A–C). Proteobacteria were observed more in the symptomatic root samples (59.60–81.80%) of Mchare than in the non-symptomatic samples (51.40–56.70%). In contrast, for Sukari Ndizi, in the non-symptomatic banana samples (60.10–67.70%) more Proteobacteria diversity was observed than in the symptomatic samples (53.50–58.20%). The members of Proteobacteria were relatively diverse and consisted mainly of Flavobacteriales, Rhizobiales, Pseudomonadales, Burkholderiales, and Firmicutes. Bacteriodetes was observed more in the non-symptomatic root samples (16.30–34.90%) of Mchare than in the symptomatic samples (10.60–19.50%). In contrast, in Sukari Ndizi, the symptomatic banana samples (7.30–23.60%) had a higher proportion of Bacteriodetes than the non-symptomatic samples did (9.20–17.10%). Among the Actinobacteria, a higher diversity was observed in the non-symptomatic (12.20–13.30%) than in the symptomatic samples (1.50–6.90%) of the Mchare cultivar. No clear differences were observed with Actinobacteria among the symptomatic and non-symptomatic samples of Sukari Ndizi. Actinomycetales and Alteromonadales were the predominant groups in the Actinobacteria.

**Figure 2:**
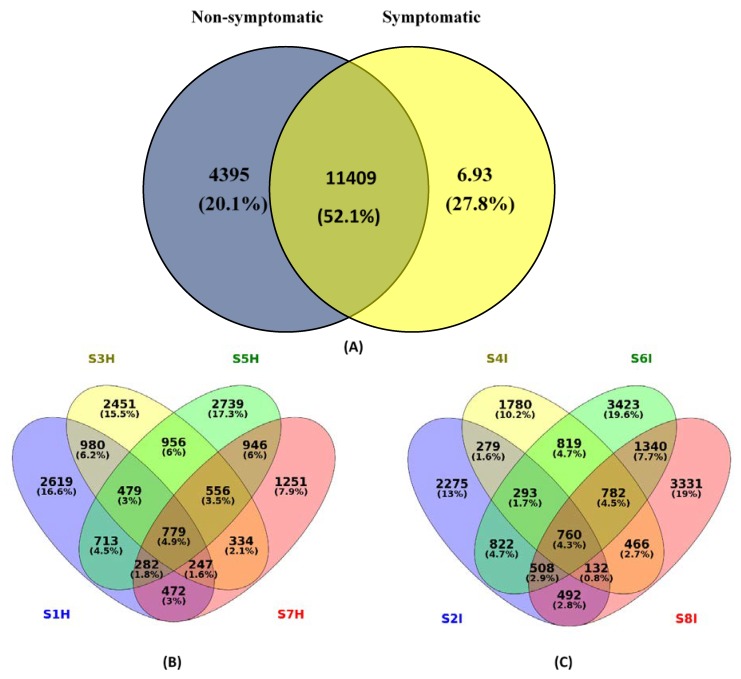
Venn diagram of the banana root microbiome: (**A**) Most abundant shared and unique OTUs in non-symptomatic and symptomatic samples, (**B**) Shared OTUs in non-symptomatic samples, and (**C**) Shared OTUs in symptomatic samples. Where: *S: Sample; 1–8: Number; H: Non-symptomatic; I: Symptomatic. The various colours corresponds to the sample location and cultivars in Venn digram. Samples from TACRI: S1H, S2I (Mchare); S3H, S4I (Sukari Ndizi) and samples from Akheri Kati: S5H, S6I (Mchare); S7H, S8I (Sukari Ndizi).

**Figure 3:**
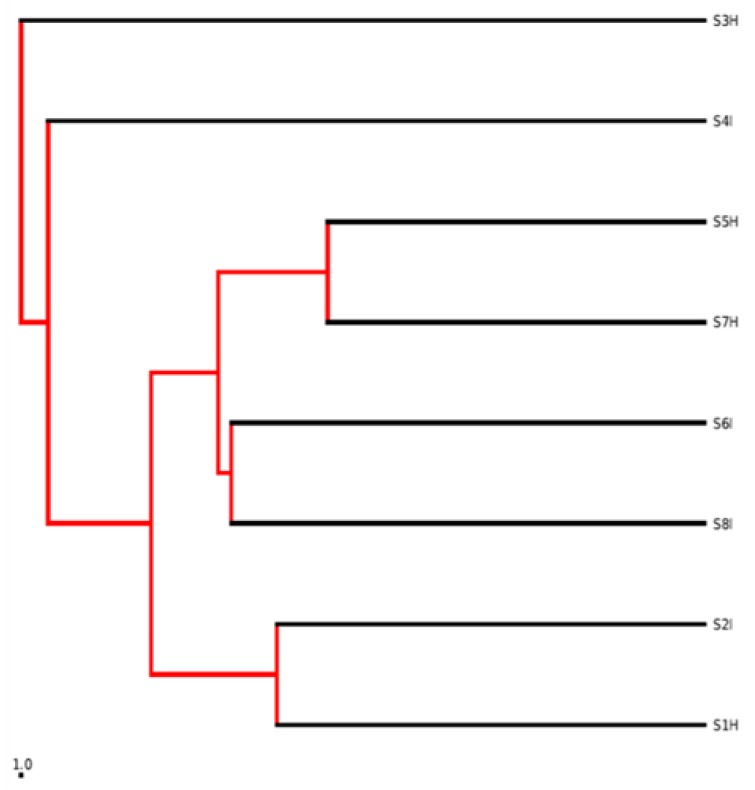
Phylogenetic map for all the root samples. Where: *S: Sample; 1–8: Number; H: Non-symptomatic; I: Symptomatic. Samples from TACRI: S1H, S2I (Mchare); S3H, S4I (Sukari Ndizi) and samples from Akheri Kati: S5H, S6I (Mchare); S7H, S8I (Sukari Ndizi).

The heatmap employing the relative abundance of the most common defined OTUs with more than 5000 sequences in the banana root endophytes displayed a significant enrichment for the OTUs belonging to Actinomycetales (mainly composed of Streptomycetales) of the phylum Actinobacteria, and Alteromonadales and Pseudomonadales of the Gammaproteobacteria. Furthermore, we analysed the most abundant 11,409 OTUs of the banana root-associated microbiome. The vast group of detected OTUs were common (52.1%); however, 20.1% were identified only in the non-symptomatic and 27.8% in the symptomatic banana root among the 11,409 that were classified as root endophytes (Figure 2). The 52.1% overlap of OTU in the Venn diagram between the symptomatic and non-symptomatic banana samples suggested that, despite the significant amount of separation in bacterial communities, there is a presence of individual bacterial OTUs among samples. Non-symptomatic cultivars of Mchare (33.90%) exhibited a higher percentage of species as compared to Sukari Ndizi (23.40%). Similarly, symptomatic samples exhibited a higher diversity in Mchare (32.60%) when compared to Sukari Ndizi (29.20%). With respect to the location, a higher number of species was observed in Akheri Kati than in TACRI, both for the non-symptomatic and symptomatic samples of both Mchare (17.3% and 19.6%) and Sukari Ndizi (7.9% and 19%), respectively. The beta diversity was calculated in a qualitative manner using Euclidean, Jaccard, and Bray-Curtis’s metrics (Supplementary Table S3). The data of the banana root-associated microbiome displayed a trend for the non-symptomatic samples of both Mchare and Sukari Ndizi cultivars irrespective of the locations. Both the symptomatic and non-symptomatic root endophytes harbored communities that are clustered together, as shown in the phylogenetic map drawn from the UPGMA analysis (Figure 3). The closeness of each root microbiome samples of the symptomatic and non-symptomatic banana plants was represented in terms of a dissimilarity matrix.

The alpha diversity of root endophytes calculated by the Shannon index displayed a significant difference and variation between symptomatic and non-symptomatic banana roots of Mchare and Sukari Ndizi (Supplementary Table S4). The root endophytes in the symptomatic plants had a higher OTU richness and diversity than in the non-symptomatic banana samples (Figure 2; Supplementary Table S4). Additional 10,809 OTUs were found in the root microbiome of bananas associated with the symptomatic samples, which revealed the presence of other microbial species in banana roots after pathogen infection. The beta diversity analysis reflected the difference in microbial composition due to the impact of the Fusarium infection of banana roots. Symptomatic samples of Mchare were dominated by Pseudomonadales (25.12%) from the samples of TACRI, and Rhizobiales and Burkholderiales (15.20%) from the samples of Akheri Kati. However, the non-symptomatic samples of Mchare were observed as having a higher diversity of Rhizobiales (28.50%) than the samples of Akheri Kati and Flavobacteriales (30.30%) from the samples of TACRI. In the case of Sukari Ndizi, a higher diversity was observed with Rhizobiales (21.10%) and Alteromonadales (18.50%) in the symptomatic samples collected from TACRI and Akheri Kati, respectively. Non-symptomatic banana samples were observed rich in Rhizobiales at both TACRI and Akheri Kati with a diversity of 26.10% and 19.50%, respectively. In general, irrespective of the location and cultivars, microbes from symptomatic banana roots were dominated by Pseudomonadales (25.12%), Rhizobiales (21.15%), Actinomycetales (19.76%), Alteromonadales (18.55%), Burkholderiales (15.22%), and Flavobacteriales (13.66%) of Proteobacteria.

It was also observed that the non-symptomatic banana roots were dominated by many specific genera, such as Flavobacterium, Pseudomonas, Devosia, and Paenibacillus.

Microbial community profiling was done in order to get a direct clue of the root-inhabiting microbiome located inside the roots and that are involved in the infection process. Proteobacteria and Bacteroidetes dominated the endophytic bacterial content of roots and accounted for approximately 92.40% of the total microbiome community. When non-symptomatic roots were compared to symptomatic roots, symptomatic roots had a higher abundance and diversity of Rhizobiales (12.85%) and Flavobacteriales (12.00%) (Figure 1C). Moreover, some OTU fractions of Flavobacteriales were also identified in one sample (S1H) of Mchare non-symptomatic banana roots collected from TACRI. Actinobacteria (mainly composed of Actinomycetales and Streptomycetales) was another predominant bacterial group, with 9.30% of the total identified OTUs among the endophytes, and it displayed a significant enrichment in symptomatic and non-symptomatic samples of Sukari Ndizi collected from TACRI. A ubiquitously identified group with a persistent community composition was Rhizobiales, for both the symptomatic and non-symptomatic roots, irrespective of the cultivars and locations.

In the present investigation, the comparative analysis of community profiles displayed a higher diversity and OTU abundance of the root microbiomes in non-symptomatic and symptomatic plants. The functional classifications revealed that 269,046 (COG database), 245,805 (KEGG pathway database), 287,043 (Pfam database), 160,806 (FIGfam database), and 173,335 (GO database) genes were annotated. The topmost taxonomic findings from the Kaiju results showed Proteobacteria with 168,867 genes as the top phylum, Alphaproteobacteria with 75,473 genes as the top Class, Rhizobiales with 53,183 as the top Order, Flavobacteriaceae with 26,166 genes as the top Family, Flavobacterium with 18,663 as the top genus, and *Fluviicola taffensis* with 8640 genes as the top species. In symptomatic samples, we identified genes encoding enzymes within microbiome that are involved in the degradation of host cell walls. Most of them were related to the oligosaccharide-degrading enzymes with few encoding endo-β-1,4-xylanase and polygalacturonase.

### 2.3. Growth Ameliorating Endophytes and Gene Function

**Figure 4:**
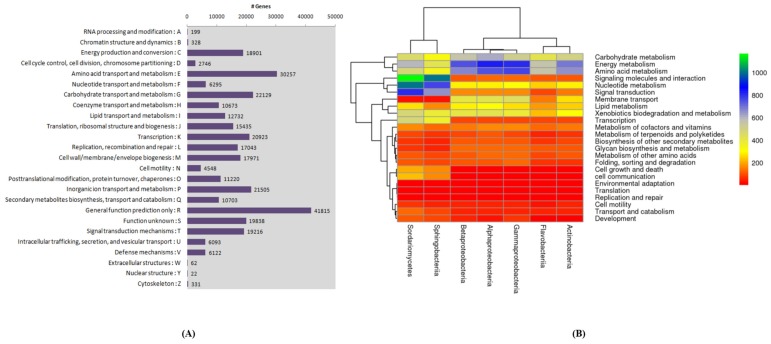
(**A**) The distribution of genes in the COG functional annotation. (**B**) Heatmap showing the relative abundance of functional categories of the major taxa-based assemblies.

The functional categories were compared to investigate the enriched functions among banana root endophytes. The COGs database was used on a phylogenetic classification of the proteins encoded in 21 complete genomes of bacteria. A different COG database was identified with the genes associated with different functional categories of the identified root microbiome (Figure 4A,B). A high relative abundance of genes with a significant functional enrichment associated with growth promotion (cell division) was observed. Several categories, including carbohydrates, protein, RNA, and DNA, which related to metabolism in plants, were observed and existed with a high proportion. Moreover, the reads that classified into virulence, disease, defense, and stress responses may contribute to disease suppression in the banana samples with *Fusarium* infection. These results were in accordance with the COG analysis mentioned above (Figure 4). Genes annotated under different functional categories also displayed a high abundance of pathogenicity-related genes (cell signaling and regulation such as quorum sensing) and membrane transport in Flavobacteriales. Apart from other endophytes, Flavobacteriales and Actinobacteriales were clustered together, displaying their close functional similarity (Figure 4B).

## 3. Discussion

Banana roots samples were collected from symptomatic and non-symptomatic banana plants of two *Fusarium* susceptible varieties, Mchare and Sukari Ndizi, from two different locations. The eight samples (S1H, S2I, S3H, S4I, S5H, S6I, S7H, and S8I) were analyzed together with a QIIME suite of tools for a microbial analysis. A metagenomic analysis of the microbial community involves problems of plant DNA intrusion which adversely affect the analysis [20,21]. In the present study, we used a well-defined protocol to eliminate the intervention of banana host DNA that inhabits *Fusarium* symptomatic roots. We clustered the similar sequences into one representative taxonomic unit called OTU. The basis of this sequence clustering was a 97% sequence similarity and was implemented through a UCLUST algorithm. These sequences were used to detect OTU in the de novo method. Following OTU detection, a representative sequence was assigned to each OTU, facilitating the taxonomic annotation using the Greengene database. Each sample of banana roots was observed with a significant difference in the community composition and species abundance. Irrespective of the cultivars and locations, Proteobacteria and Actinobacteria were consistently enriched in the roots of both symptomatic and non-symptomatic samples, as observed by other researchers for the plant species of banana, rice, maize, wheat, and sugar cane [22,23,24], which are generally known for their good response to labile carbon sources with the fast growth of the plants [25,26]. Taken together, our results reveal that Proteobacteria and Actinobacteria are adapted to banana roots. Further analyses of most abundant banana-associated OTUs in root samples reflect a community variation with OTUs’ quantity and not their species in both Mchare and Sukari Ndizi cultivars. These most abundant OTUs of endophytes identified in the roots are consistent with earlier reports which further hypothesize that the endophytic microbiome was acquired from Rhizobacteria [5].

The host-microbiome interactions might change when a plant is attacked by phytopathogens. We found a significant fraction of variation and differentiation in the endophytic diversity (both α- and β-diversity) from the infection by *Fusarium* in both Mchare and Sukari Ndizi cultivars. This could be attributed to the host genetics of symptomatic samples when compared to those from non-symptomatic banana roots. More dispersion of OTUs among the symptomatic samples displayed that other microbes were present in the root samples associated with pathogen infections. In the non-symptomatic roots of Mchare and Sukari Ndizi cultivars, Rhizobales and Flavobacteriales were present in very small fractions (less than 1%), but a significantly enhanced fraction (from 12% to 21%) was noticed in the symptomatic root microbiome of both cultivars. The different root microbiome of symptomatic and non-symptomatic samples is mostly due to variation in the members of Proteobacteria (observed in Mchare and Sukari Ndizi), as confirmed by a paired metastatistical analysis.

In the non-symptomatic banana root microbiome of Sukari Ndizi (16.30%) and Mchare (12.00%) cultivars, Rhizobiales are associated with a high abundance of the diverse group of symbiotic nitrogen-fixers *Devosia*. Samples collected from non-symptomatic banana roots from Akheri Kati of Mchare and Sukari Ndizi cultivars also exhibited a lower but significant abundance of *Bradyrhizobium*, at 3.70% and 4.60%, respectively. However, *Agrobacterium*, a very common plant pathogen, was the predominant bacterial group (2.20–5.30%) present with Rhizobiales in both cultivars at both locations. Producers of active metabolic compounds against phytopathogens such as Actinomycetales were also present in the root microbiome [27,28] and predominant in the non-symptomatic (6.70%) and symptomatic (14.90%) samples of Sukari Ndizi. This could demonstrate the biocontrol response against *Fusarium* infection in bananas via the variation in the microbial community between non-symptomatic and symptomatic plant roots. Our work is supported by the previous study on Pseudomonadales, the third-most abundant bacterial group in non-symptomatic banana roots. A high abundance of Pseudomonadales was observed in both the Mchare (16.80%) and Sukari Ndizi (7.50%) cultivars of non-symptomatic banana root samples, as compared to symptomatic samples. Pseudomonadales has been widely investigated for its growth promotion and phytopathogen suppression efficacies, and also in order to induce systemically induced resistance in plants [29,30]. Variation in the composition and abundance of the endophytic microbial community in symptomatic root samples provides the clue for the effective response by beneficial microbes in root tissues during fungal pathogen attacks.

Endophytes have multiple strategies to overcome competition. Our results are supported by the microbial community analysis of banana, sugar cane, and rice plants, which harbored an identical microbiome profile dominated by Actinobacteria and Proteobacteria groups [31,32,33]. Before attacking plants, phytopathogens secrete a number of lytic enzymes (pectinase, xylanase, and cellulase) in order to break the cell wall, which is mainly composed of lignocellulose and in order to potentially protect the plant from invasion by foreign microbes [34]. Endophytes also break the lignocellulosic barrier for their entry but do so without causing any negative symptoms in the host tissue, as the bacterial entry into the root tissue of hosts is the pre-requisite to become an endophyte [35]. It is also evident that the endophytic colonization in host root tissues involves cell wall degradation enzymes such as pectinases and cellulases such as endoglucanase and polygalacturonase, which are responsible for bacterial entry and colonization [36]. The native endophytic microflora of plant root tissue might secrete various enzymes for cell wall penetration that enable the outsider microflora to reside and replicate within the host. To analyse the endophytic capabilities to produce lytic enzymes, we investigated an assembly of metagenomic data for the existence of gene sequence codes for lignocellulose-degrading enzymes. The functional annotation was done with COGNIZER to analyse the number of genes assigned an annotation from each type of metagenomic database viz., COG, Pfam, FIG, KEGG, GO, GenBank, PATRIC, and eggNOG. The presence of genes associated with oligosaccharide degradation elucidated that endophytes degraded a small amount of polysaccharides (plant-associated) and used oligosaccharides as a nutrient source with no damage or negative impact on the host tissue [36].

Endophytes have been widely studied in relation to benefitting plant health through various kinds of growth promoting, disease suppression, and RIDER mechanisms [30]. The diversity of microbiota associated with roots involved complex plant-microbe interactions which are crucial for plant health. In our study of the metagenomic analysis of the banana root microbiome, we identified endophytes like *Bacillus* and *Pseudomonas* in the non-symptomatic root samples of Sukari Ndizi and Mchare cultivars. The endophytes like *Bacillus* have been effectively proven to possess a large number of plant-growth promoting (nutrient solubilization and uptake, production of phytochromes) and disease control (acting as biocontrol agents) attributes. Endophytes are widely known to produce IAA (indole-3-acetic acid) to promote plant growth and development [37,38]. In addition to the diversity, the taxonomic composition revealed a predominant colonization by Pseudomonadales (in both Mchare and Sukari Ndizi), which are known plant colonizers (particularly the genus *Pseudomonas*) and are responsible for beneficial plant-microbe interactions [39]. The metagenomic analysis of banana root endophytes also revealed that it could be possible to get a potential candidate which serves as a biocontrol agent against Fusarium wilt and other phytopathogens in bananas. The abundance of Pseudomonadales was observed as showing a drastic increase (24.70%) in the symptomatic samples of Mchare collected from TACRI. In the meantime, the high frequency presence of alpha-, beta-, gamma-, and delta-Proteobacteria groups in Proteobacteria in symptomatic samples could be considered as an important bacterium in the degradation of all kinds of glucoses, and could partially contribute to the symptom observed after *Fusarium* infection [40]. Finally, all these enriched root microbiomes advocated that the root endophyte microbiotas are selected based on the innate immune system of the plant. Some of them might have performed as pathogenic complexes during the infection of the host as it was also evident that non-antagonistic microbial strains can become antagonistic when they grow with other specific strains [41].

The taxonomic classification and functional annotation in this study were carried out using different pipelines. Using comparative pipelines made our results more reliable and allowed us better chances to explore information that may otherwise have been left undiscovered.

## 4. Materials and Methods

### 4.1. Sample Collection and DNA Extraction

**Figure 5:**
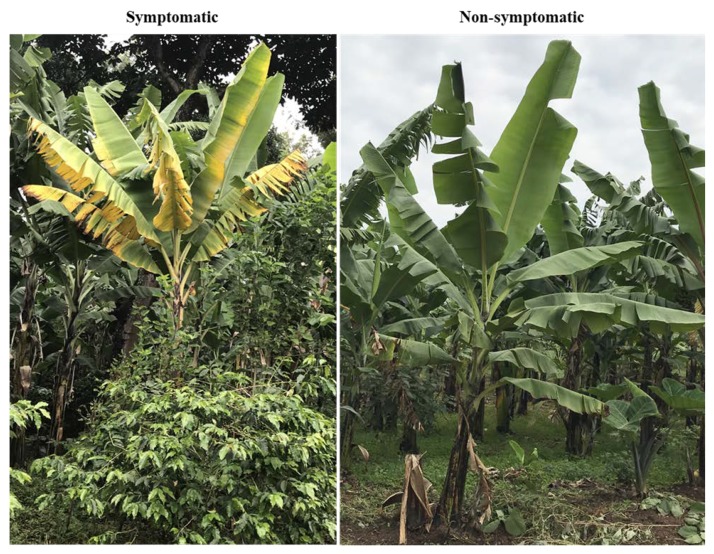
Banana plants used for sampling with typical symptoms of Fusarium wilt and with no symptoms.

Banana root samples were collected from TACRI (N″ 36.4821°; E″ 3.235°; 1230 m), in the Kilimanjaro and Akheri Kati (N″ 36.77541°; E″ 3.35579°; 1452 m), in Arusha, Tanzania. Samples were collected from Mchare and Sukari Ndizi cultivars from eight year old plants targeting Foc symptoms and non-symptoms (lack Foc symptoms in plants) in the same field (Figure 5). 

Both the cultivars are susceptible to Foc infection and are widely grown by smallholder farmers in the region. Root samples were collected from a 15–30 cm depth from three random locations around a single plant and were directly attached to the corm. For each cultivar (Mchare and Sukari Ndizi) and type (Foc symptomatic and non-symptomatic), three plants (each from five different fields) were targeted for the collection of root samples and made as one composite sample. In total, we collected eight composite samples from two different sites representing non-symptomatic (S1H, S3H, S5H, and S7H) and symptomatic (S2I, S4I, S6I, and S8I) bananas (Supplementary Figure S1). The samples were immediately transferred to polythene bags and kept in ice until arrival at the laboratory. The samples were kept at 4 °C until processed. DNA was extracted using the Plant DNA Isolation Kit (Zymo Research Corp., Irvine, CA, USA).

### 4.2. PCR Amplification of 16S rRNA Gene

The primers 460F (5′-CCTACGGGNBGCASCAG-3′) and 460R (5′-GACTACNVGGGTATCTAATCC-3′) were used to amplify the V3-V4 hyper-variable region of the 16S rDNA gene of bacteria and archaea [42]. The amplicons were amplified using i5 and i7 primers as per the standard Illumina protocol [42]. The amplicon library was prepared with Nextera XT Index Kit (Illumina Inc., Hayward, CA, USA) as per the 16S Metagenomic Sequencing Library preparation protocol with a 2 × 150 read length. The amplicon libraries were purified by 1X AMpureXP beads, checked on Agilent DNA1000 chip on Bioanalyzer2100, and quantified by Qubit Fluorometer 2.0 using Qubit dsDNA HS Assay kit (Life Technologies, Carlsbad, CA, USA).

### 4.3. Bioinformatic Analysis of 16s Results

High quality paired-end reads were first stitched using a FLASH tool with a minimum of 10 bp overlap to recreate the V3-V4 (forward and reverse hypervariable) regions and were then taken for analysis. Furthermore, chimeras generated during the PCR process were first removed, using the usearch61 algorithm. Then, similar sequences were clustered using the UCLUST algorithm and brought together into one representative taxonomic unit called the Operational Taxonomic Unit (OTU) at 97% sequence similarity criteria.

### 4.4. Sequencing, Assembly and Binning of the Pathogenesis-Associated Metagenome

The paired-end sequencing library was prepared with Truseq Nano DNA Library Prep kit (Cat. No. 20015965). The library preparation process for all the eight samples (four symptomatic and four non-symptomatic) was initiated with 200 ng of gDNA [43].

### 4.5. Metagenome Assembly and Gene Prediction

The de novo assembly of high quality paired-end reads was accomplished with metaSPAdes at default parameters including auto kmer. The genome assembler of metaSPAdes is a de Bruijn graph-based assembler that performed well for metagenome data [43]. The statistical elements (number of scaffolds, size of scaffolds, maximum and minimum size of scaffold, and their N50 value) of the assemblies were calculated by Perl scripts. These generated scaffolds were subjected to genes prediction with Prodigal (v2.6.3) at the metagenome mode and at default parameters. These predicted genes were then taken further for a taxonomic and functional analysis.

### 4.6. Taxonomic Classification

The high-throughput sequencing data were classified from the whole metagenome sequencing with the Kaiju program. First, the sequences were translated into the six possible reading frames, and the resulting amino acid sequences were split into fragments at their stop codons. The fragments were then sorted by their BLOSUM62 score (Greedy mode). This sorted list of fragments was then searched against the reference protein database (NCBI non-redundant) with the backwards search algorithm on the BWT (Burrows–Wheeler Transform algorithm). Once the remaining fragments in the list could not achieve a better score (Greedy), the search stopped, and the taxon identifier of the corresponding database sequence was retrieved.

### 4.7. Functional Annotation

The functional annotation was done with COGNIZER against a multiple database. COGNIZER (v0.9b) was used at default parameters to assess the functional capacities of the microbial communities, and to simultaneously provide Clusters of Orthologous Groups of proteins (COGs), KEGG (Kyoto Encyclopedia of Genes and Genomes), Pfam, GO (Gene Ontology) and FIGfams annotations to individual sequences. The predicted genes were taken as an input in COGNIZER for assigning annotations using multiple databases.

### 4.8. Annotation from COG Database and KEGG

The COG database is an attempt for a phylogenetic classification of the proteins encoded in 21 complete genomes of bacteria, archaea, and eukaryotes. The COGs were constructed by applying the criterion of consistency of genome-specific best hits to the results of an exhaustive comparison of all protein sequences from these genomes. This annotation aids in relating the identified genome to other bacterial taxonomic lineages for both functionality and phylogeny. Genes annotated with different functional attributes were displayed under different orthologous groups of the COG database. The KEGG database was used to associate metabolic pathways with respective genes. The functional heatmap showed a taxonomic assignment for each OTU (operational taxonomic unit).

### 4.9. Nucleotide Sequence Accession Numbers

The sequence data were deposited in NCBI with SRA accession PRJNA493728 and PRJNA494072.

## 5. Conclusions

In the present investigation, root-associated microbiomes were investigated in symptomatic and non-symptomatic banana plants with the aim to investigate the responses of the endophytic community during *Fusarium* infection and their functional attributes in endophyte-plant-fusarium interactions. In our study, the non-symptomatic plants might have been infected due to the nature of Foc that persists in soil for decades, even without a host and without the movement of farmers and equipments between farms. However, the non-symptomatic plants were showing no symptoms of disease development. Furthermore, Proteobacteria, followed by Bacteriodetes and Actinobacteria, were the predominant bacterial groups. A comparative community analysis of non-symptomatic and symptomatic banana roots also revealed the presence of Pseudomonadales and Streptomycetaceae, widely known to produce antagonist compounds against phytopathogens. The manipulation of pseudomonads and streptomyceae populations in rhizosphere soils could help us to reduce/diminish the disease development in plants, even in the presences of the pathogen. Root microbiomes of both Mchare and Sukari Ndizi cultivars were observed with an increased abundance of Flavobacteriales and Rhizobiales endophytic groups, known to be involved in the carbohydrate metabolism. A metagenomic analysis also revealed that several bacterial communities are associated with key infection processes that include symbiotic relationships for the sake of nutrients, cell wall destruction at potential entry sites of pathogens, as well as infection processes in banana roots. Future studies will focus on the investigation of root pathogenesis-associated microbial communities, including activities and functions of the microbiome, in order to understand complex microbe-plant-pathogen interactions that will provide new opportunities to understand how microbiomes control plant health and that will open new avenues to increase crop production.

## Supplementary Materials

The following are available online at https://www.mdpi.com/2223-7747/9/2/263/s1, Figure S1. Layout plan for the banana root sample collection. Table S1. Summary of the QIIME filtration and chimeric sequences removal. Table S2. Summary of clustering the similar sequences into one representative taxonomic unit as the Operational Taxonomic Unit (OTU). Table S3. Summary of the beta diversity for each sample. Table S4. Summary of the alpha diversity for each sample.
