# Supplementary material for: Metagenomic Insights of the Root Colonizing Microbiome Associated with Symptomatic and Non-Symptomatic Bananas in Fusarium Wilt Infected Fields
Source: Plants (Basel). 2020 Feb 18;9(2):263. doi: 10.3390/plants9020263 (PMC7076721; doi:10.3390/plants9020263)
Supplement: Supplementary file 1 [file plants-09-00263-s001.pdf]

Title: Metagenomic insights of the root colonizing microbiome associated with symptomatic and non-symptomatic bananas in *Fusarium* wilt infected fields

Manoj Kaushal[1], George Mahuku[1], Rony Swennen[2,3,4]

[1]International Institute of Tropical Agriculture (IITA), Mikocheni B, Dar es Salaam -34441, Tanzania. [2]Bioversity International, Willem De Croylaan 42, B-3001 Leuven, Belgium

[3]Laboratory of Tropical Crop Improvement, Division of Crop Biotechnics, KU Leuven, B-3001 Leuven, Belgium

[4]International Institute of Tropical Agriculture. c/o The Nelson Mandela African Institution of Science and Technology (NM-AIST), P.O. Box 447, Arusha, Tanzania

Corresponding author email: M.kaushal@cgiar.org

Supplementary Figures:

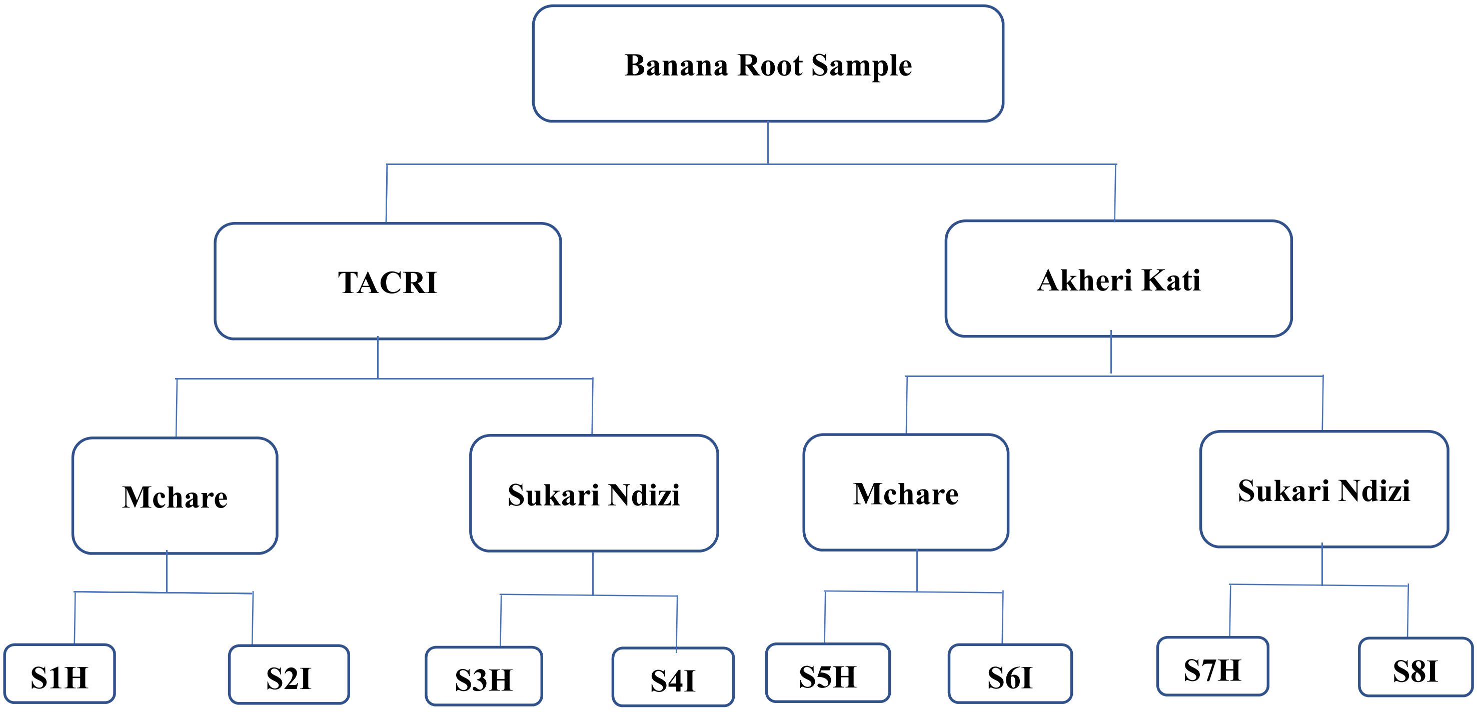

Figure S1. Layout plan for banana root sample collection. ** A total of eight banana root samples and each sample is a composite of three sub-samples.

Where: *S: Sample; 1-8: Number; H: non symptomatic; I: symptomatic

Supplementary Tables

Table S1 Summary of QIIME filtration and chimeric sequences removal

Sample	# of Stitched Reads	# non chimeric Reads
S1H	349670	308784
S2I	381417	337074
S3H	278606	242504
S4I	307503	281162
S5H	544149	464508
S6I	457699	385006
S7H	222216	199861
S8I	467607	414864

Table S2 Summary of clustering the similar sequences into one representative taxonomic unit as Operational Taxonomic Unit (OTU)

Sample	# OTUs found	# OTUs found (excluding singleton OTUs)
S1H	15426	6571
S2I	11779	5561
S3H	14909	6782
S4I	10044	5311
S5H	16699	7450
S6I	19223	8747
S7H	8243	4867
S8I	15901	7811
total collective OTUs	81021	21897

Table S3 Summary of Beta diversity for each sample

Table S4 Summary of alpha diversity for each sample

Sample	Shannon	Observed_species	Chao1
S1H	6.88506523	6571	9800.60656
S2I	6.37236815	5561	8143.76482
S3H	7.08072462	6782	10410.5758
S4I	6.96542793	5311	7038.07692
S5H	7.3710425	7450	10055.5672
S6I	7.81189461	8747	11979.4332
S7H	7.58508533	4867	7742.43584

Abundance jaccard

S3H	S7H	S4I	S6I	S8I	S5H	S1H	S2I	
**S3H**	0	0.38994654	0.40508105	0.29657489	0.356525	0.24514122	0.32363247	0.26662991
**S7H**	0.38994654	0	0.22350251	0.21517002	0.32101784	0.19348237	0.31286512	0.24998679
**S4I**	0.40508105	0.22350251	0	0.23322821	0.38825299	0.18285739	0.32431332	0.29862069
**S6I**	0.29657489	0.21517002	0.23322821	0	0.30214918	0.22757668	0.32953169	0.27349194
**S8I**	0.356525	0.32101784	0.38825299	0.30214918	0	0.35111298	0.41176137	0.39858321
**S5H**	0.24514122	0.19348237	0.18285739	0.22757668	0.35111298	0	0.31394417	0.22663904
**S1H**	0.32363247	0.31286512	0.32431332	0.32953169	0.41176137	0.31394417	0	0.1894764
**S2I**	0.26662991	0.24998679	0.29862069	0.27349194	0.39858321	0.22663904	0.1894764	0

Bray curtis

**S3H**	0	0.72755078	0.77403536	0.76482925	0.72336347	0.69802776	0.79598685	0.76477368
**S7H**	0.72755078	0	0.60576106	0.57402281	0.67239985	0.65609172	0.69109693	0.75519569
**S4I**	0.77403536	0.60576106	0	0.68109846	0.79915693	0.63907895	0.82348215	0.85522681
**S6I**	0.76482925	0.57402281	0.68109846	0	0.6615375	0.72413874	0.70794621	0.72667018
**S8I**	0.72336347	0.67239985	0.79915693	0.6615375	0	0.77044527	0.743378	0.76228891
**S5H**	0.69802776	0.65609172	0.63907895	0.72413874	0.77044527	0	0.72264552	0.76687101
**S1H**	0.79598685	0.69109693	0.82348215	0.70794621	0.743378	0.72264552	0	0.56595103
**S2I**	0.76477368	0.75519569	0.85522681	0.72667018	0.76228891	0.76687101	0.56595103	0

Euclidean

**S3H**	0	48245.8342	67070.1317	71066.4487	66133.1793	63376.7844	65445.5973	74114.055
**S7H**	48245.8342	0	46510.1299	52123.5513	57440.6802	52529.2641	45892.8103	60569.3758
**S4I**	67070.1317	46510.1299	0	70761.298	81654.3912	62362.3777	69127.9871	84169.1469
**S6I**	71066.4487	52123.5513	70761.298	0	63363.7117	75884.0311	63475.5343	73382.7122
**S8I**	66133.1793	57440.6802	81654.3912	63363.7117	0	79203.728	64039.8748	74062.3685
**S5H**	63376.7844	52529.2641	62362.3777	75884.0311	79203.728	0	62680.0071	74408.7829
**S1H**	65445.5973	45892.8103	69127.9871	63475.5343	64039.8748	62680.0071	0	47359.2836
**S2I**	74114.055	60569.3758	84169.1469	73382.7122	74062.3685	74408.7829	47359.2836	0

S8I 7.61245204 7811 10539.395
